# Protective Impacts of *Moringa oleifera* Leaf Extract against Methotrexate-Induced Oxidative Stress and Apoptosis on Mouse Spleen

**DOI:** 10.1155/2020/6738474

**Published:** 2020-05-29

**Authors:** Mohamed Mohamed Soliman, Saad Hommod Al-Osaimi, Essam HassanMohamed, Adil Aldhahrani, Adel Alkhedaide, Fayez Althobaiti, Wafaa Abdou Mohamed

**Affiliations:** ^1^Clinical Laboratory Sciences Department, Turabah University College, Taif University, Taif 21995, Saudi Arabia; ^2^Biochemistry Department, Faculty of Veterinary Medicine, Benha University, Benha 13736, Egypt; ^3^Chemistry Department, Turabah University College, Taif University, Taif 21995, Saudi Arabia; ^4^Department of Biology, Turabah University College, Taif University, Taif 21995, Saudi Arabia; ^5^Department of Microbiology, Faculty of Veterinary Medicine, Zagazig University, Zagazig 44519, Egypt; ^6^Department of Biotechnology, College of Science, Taif University, Taif, Saudi Arabia; ^7^Clinical Pathology Department, Faculty of Veterinary Medicine, Zagazig University, Zagazig, Egypt

## Abstract

**Objective:**

The current study was aimed to examine the possible ameliorative impacts of MO leaf extract (MOLE) against MTX-induced alterations on oxidative stress of mouse spleen and explore the possible molecular mechanism that controls such impacts.

**Methods:**

Adult male mice were allocated into 4 groups: control, *Moringa oleifera* leaf extract (MOLE), MTX, and MOLE plus MTX. Mice received MOLE orally for a week before MTX injection and continued for 12 days. Serum and spleen were sampled for biochemical and quantitative gene expressions.

**Results:**

As compared with the MTX-injected group, MOLE effectively reduced the changes in total proteins, spleen MDA, SOD and catalase activities, and changes in serum antioxidants levels. Moreover, there is downregulation of antioxidant genes (SOD and catalase) and antiapoptotic genes (XIAP and Bcl-xl) along with upregulation in Bax and caspase-3 mRNA (apoptotic genes) in the MTX-injected group. MTX induced changes in IL-1*β*, IL-6, TNF-*α*, and IL-10 expression. MOLE restored and ameliorated the changes induced in biochemical, antioxidants, apoptosis, and apoptosis associated genes that were induced by MTX intoxication.

**Conclusion:**

Current findings indicated that pretreatment with MOLE to MTX-intoxicated mice showed the potential usage of MO for oxidative stress and apoptosis treatment.

## 1. Introduction

Alternative medicine of herbal origin is widely used all over the world for treatment of toxicity and its associated disorders [1, 2]. Herbal medicines act as ant-antioxidants and free radical scavengers for all organs. They induced organ regeneration and protection to improve their vitality and stability [2, 3]. The use of herb extracts in alternative medicine is increased, and the presence of many plants with the medicinal reference became conventional in therapeutic medicine. *Moringa oleifera* (MO) is growing in many countries (in tropical and subtropical) and is highly nutrient-rich plant with pleiotropic medicinal properties [4].

*Moringa oleifera* leaves (MOL) and its seeds are very rich with antioxidants, vitamins (A, D E, C, and *γ*-carotene) [4], and polyphenols [5]. Moringa leaves are rich in amino acids, carbohydrates, fats, minerals, and vitamins [5]. Previous studies confirmed the potential therapeutic potencies of *Moringa oleifera* as antidiabetic, antifungal, antimicrobial [6], and antiatherosclerotic agents [7]. Moreover, MO has the potential in regulation of thyroid function against oxidative stress and damage [5, 7] and enhances sexual performance in rats under stress [8]. The consumption of MO is safe, and its toxicity rate is very low [9]. Therefore, when MO is consumed in a higher quantity, it showed nontoxic events [10].

Lake of proportion between antioxidants and pro-oxidants shifts towards the pro-oxidant state which is the cause for oxidative stress that is known as reactive oxygen species (ROS). ROS is produced from local or external sources as methotrexate (MTX) and chemical-intoxicated materials [11, 12]. ROS plays a critical role in normal physiological processes [12] but excess of ROS can damage cell structures that can contribute to disease [13]. Therefore, organs and tissues are adapted to protect their cells against ROS and oxidative stress to maintain proper redox homeostasis against excess ROS [13].

The folate antagonist, methotrexate (MTX), is used as chemotherapeutic drug for treatment of cancer [14, 15]. However, MTX usages in therapy show some toxic effects [16]. The mechanism by which MTX-induced toxicity is not yet completely examined [17]. The MTX toxicity on internal organs such as the kidney, liver, and heart is mainly attributed to the oxidative stress induction [18–20]. Till now, there is no clear study about the direct toxic effects of MTX on spleen. However, MTX still the most-effective chemotherapeutic drug used in medication. For that, physician efforts to prevent and overcome MTX side-effects must be directed. The properties and function of *Moringa oleifera* (MO) have been discussed by several papers; however, till now no clear study shows the protective impacts of MOLE against MTX-stimulated splenic oxidative stress and apoptosis.

Spleen plays critical supporting roles in the homeostasis of body. It acts as a barrier and filter for blood as part of the immune system. It is the most critical organ affected by stress; however, no more data and reports are available about it during oxidative stress. Therefore, the current study examined the protective impact of MOLE against oxidative stress and apoptosis in the spleen of male mice induced by MTX.

## 2. Materials and Methods

### 2.1. Chemicals and Measurements

Ethidium bromide, methotrexate, and agarose were purchased from Sigma-Aldrich, St. Louis, MO, USA. The reverse transcriptase and the 100 bp ladder (DNA marker) were bought from MBI, Fermentas, Thermo-Fisher Scientific, USA). Oligo dT primers and Qiazol for total RNA extraction were imported from QIAGEN, Valencia, CA, USA. The kits for catalase, GSH (reduced glutathione), SOD (superoxide dismutase), MDA, total proteins, albumin, and globulin were from Biodiagnostic Co. (Dokki, Giza, Egypt). The ELISA kits for Mouse IL-1*β* (ab197742) and IL-6 (ab100712) were bought from Abcam Co., Tokyo, Japan. IL-1*β* and IL-6 were measured as described in the protocol manual of each kit.

### 2.2. Preparation of *Moringa oleifera* Leaf Extract (MOLE)

The fresh leaves of *Moringa oleifera* (MOL) were identified by a botanist (Prof. Yassin Alsudani) at the College of Science, Taif University. Voucher specimens have been deposited in the PANDA herbarium at Hawiya, Taif, Saudi Arabia. MOL were dried under room temperature at 29°C in dark, after which the leaves were pulverized with a crestor high-speed milling machine. MOL were immersed in hydroalcoholic solution (40% distilled water + 60% ethanol) in a stoppered flask and was kept at room temperature for 48 hrs at 100 rpm in an orbital shaker. The extract contents were filtered and passaged on whatman paper #1 [21]. The resulting hydroalcoholic filtrate was concentrated and evaporated to dryness using a rotary evaporator (Rotavapor® R-300/R-300 Pro, https://www.buchi.com/rotavapor-r-300) at 42°C to avoid denaturation of the active ingredients. The residue yield was 12% (w/w) and kept at −20°C till use.

### 2.3. Total Polyphenolic Compound Estimation, Chemical Composition, and Ultraviolet-Visible (UV-Vis) Spectroscopy of MOLE

The total polyphenolic compounds of MOLE were evaluated by Foline-Ciocalteu reagent [22] and modified by others [23, 24]. The total polyphenolic ingredients were shown as milligrams of gallic acid equivalent per 100 grams of MOLE. The standard curve used was gallic acid at different concentrations (25, 50, 100, 150, and 200 *μ*g/ml) to prepare the standard curve. For each standard concentration and sample, the absorbance at 760 nm was measured, and the calibration curve was obtained and calibrated. The chemical composition of MOLE was assayed as stated before [25]. Total phenolic ingredients of MOLE were measured in UV-Vis spectrum of the reaction mixture at *λ* 200–400 nm after 10 folds dilution for each sample with deionized water. The UV-Vis spectroscopy was done using UV-Vis spectrophotometer UV-240.

### 2.4. Animals, Sampling, and Experimental Design

**Figure 1:**
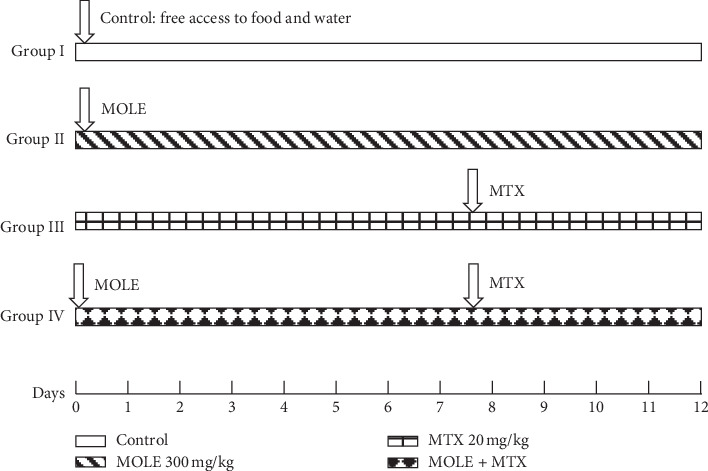
A Schematic diagram showing the experimental design. MOLE: *Moringa oleifera* leaf extract; MTX: methotrexate.

Experimental mice were from College of Pharmacy, King Abdel-Aziz University, Saudi Arabia). A total of 28 male mice aged 10 weeks, with weight of 40 grams, were used for current study. To ensure mice adaptation and to avoid handling stress, mice were handled manually for 10 days. The ethical committee of Turabah University approved the procedures used in the current study. The animals were housed in the laboratory house of Turabah University College with free access to food and water at 25 ± 5°C. Mice were free of any disease and were allocated into 4 groups:

  Group I: served as negative control (CNT) with free access to water and food.
  Group II: *Moringa oleifera* leaf extract (MOLE) administered orally in a dose of 300 mg/kg bw daily for consecutive 12 days [21]. The dose used here is the most effective dose that treats organ toxicity and reported by other papers.
  Group III: positive intoxicated group, MTX administered intraperitoneally (IP) at a dose of 20 mg/kg bw, once on day 7. For MTX, the timing and optimal dosages were determined based on reported studies [17, 19]. MTX dose (20 mg/kg bw IP) was nearly the same reported for humans. Therefore, MTX in a dose of 20 mg/kg is the optimal high dose that induces organ toxicity in animals [19].
  Group IV: MOLE administered orally for one week, and on day 7, MTX was injected and continued with MOLE for another 5 days. The schematic diagram for the current experimental protocol is illustrated in Figure 1.

MOLE administration for a week prior to MTX injection is enough and fit to stimulate Th1/Th2 cytokines. Therefore, 5 days after injection of MTX is proper time to check the recovery from alterations induced by MTX toxicity in spleen [26]. At the end of study design, mice inhaled dimethyl ether, blood was collected, and then mice were decapitated for tissue sampling. Serum was extracted from blood by centrifugation at 4000 rpm for 6 min and stored in the freezer at −20°C till biochemical measurements. The splenic tissues were immersed either in Qiazol (Qiagen Co, USA) for extraction of RNA or in ice phosphate buffer for splenic antioxidants measurements.

### 2.5. Preparation of Spleen Homogenate and Oxidative Stress Biomarker Measurements

Spleen tissues were homogenized (10% w/v) in 0.1 M phosphate buffer (pH 7.4) using a sonicator (4710 Ultrasonics Homogenizer, Cole- Parmer Instrument Co., USA) [27]. The homogenate was centrifuged at 5000 rpm for 5 min at 4°C. The supernatant layer was taken and kept at −20°C for measurements of MDA, GSH, and SOD. The MDA, GSH, and SOD activities in the homogenate were assayed using the ELISA reader (Bio-Rad Co., NY, USA), following the procedure written in the product instruction manual with reference to the previous reported studies [28–30].

### 2.6. Biochemical and Antioxidant Measurements

The serum samples were collected and examined for the changes in antioxidants and oxidative stress biomarkers. Lipid peroxidation (LPO; malondialdehyde (MDA)) was measured based on the method reported before [28]. The activity of superoxide dismutase (SOD), reduced glutathione (GSH) content, and catalase was measured based on the previous established reports [29–31]; respectively. The total proteins were measured in serum samples based on method of Lowry et al. [32]. Albumin and globulin levels were measured as stated before [33].

### 2.7. Semiquantitative RT-PCR and Gene Expression

Total RNA was extracted from the spleen of all mice [34]. RNA integrity was confirmed in denatured gel. For RT-PCR, 3 *μ*g of RNA was reverse transcribed using oligo dT primer at 70°C for 5 min for denaturation in a PCR thermal cycler machine (Bio-Rad T100TM). A mixture of 2 *μ*L 10X RT-buffer, 2 *μ*L of 10 mM dNTP, and 1 *μ*L of 100 MUL-reverse transcriptase in a total volume of 20 *μ*L was used for cDNA synthesis after incubation at 42°C for 1 hr and then incubated at 70°C for 10 min to ensure enzyme inactivation. The primers shown in Table 1 were used for PCR reaction. PCR genes and reactions are shown in Table 1 in a total volume of 25 *μ*L using 2X Master Mix (Promega Corporation, Madison, WI, USA). The expression of housekeeping gene, *β*-actin, was used to check sample stability and quantify the examined genes. PCR products were run on stained (ethidium bromide) agarose gel (1.5%) and then visualized and copied using the gel documentation system. The band intensities were quantified using Image J software (Version 1.47) (https://imagej.nih.gov/ij/index.html).

### 2.8. Statistical Analysis

Results are shown as mean ± standard error of means (SEM) for seven different mice for each group. One-way ANOVA and Dunnett's post hoc descriptive test analyzed the current data using SPSS software for Windows (SPSS, IBM, Chicago, IL, USA). Values with means of *P* < 0.05 were statistically considered significant.

## 3. Results

### 3.1. Analysis and Detection of MOLE Ingredients

**Figure 2:**
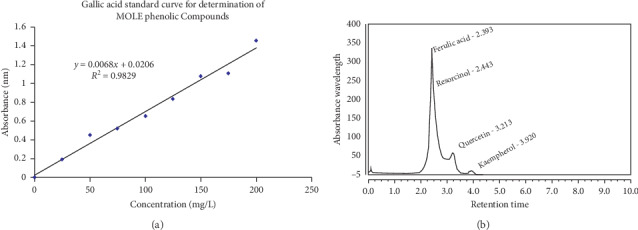
Gallic acid standard curve (a) and total polyphenolic compounds of MOLE (b).

MOLE based on used gallic acid standard curve (Figures 2(a) and 2(b) and Table 2) revealed the presence of 4 major polyphenolic compounds in MOLE (ferulic acid 61.09%, quercetin 21.18%, resorcinol 7.46%, and kaempherol 6.18%). The composition of MOLE showed that it contains ash 11%, moisture 5.99%, crude fiber 9.91%, crude fat 10.7%, crude protein 22.1%, and carbohydrates 49.37%, respectively. The total polyphenolic compounds were 65–75 mg/100 grams of moringa leaves.

### 3.2. Impact of MOLE on Oxidative Stress in Splenic Tissue Homogenate

MTX-injected mice (Table 3) showed an increase in tissue damage that was represented by an increase in MDA levels and a decrease in GSH and SOD activities in spleen homogenates. MOLE alone showed good antioxidant activity. When MOLE was preadministered for a week before MTX intoxication and continuation for 5 days, it protected the changes in MDA, GSH, and SOD levels in the spleen altered by MTX intoxication (Table 3).

### 3.3. Changes in Serum Antioxidants and Biomarkers of Oxidative Stress

**Figure 3:**
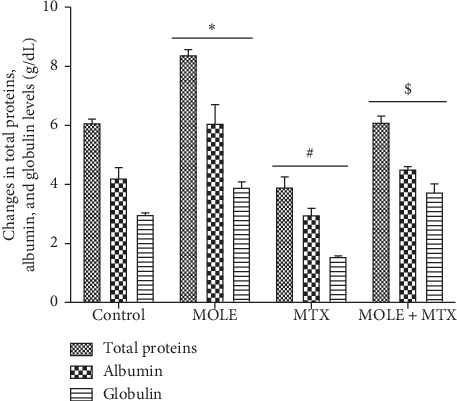
Ameliorative effects of MOLE against MTX-induced changes in total proteins, albumin, and globulin. Values are statistically significant at ^*∗*^*P* < 0.05 versus control; ^#^*P* < 0.05 versus control and moringa groups; and ^$^*P* < 0.05 versus MTX group.

Table 4 shows SOD, GSH, and catalase activities in the serum of the MTX-injected mice. The levels of SOD, GSH, and catalase were significantly decreased compared with that of control and MOLE administered groups. Preadministration of MOLE to the MTX-injected group resulted in recovery in all examined enzymatic activities to normal levels (Table 4). Unlike the decrease in antioxidants levels, the levels of MDA in the serum of MTX-injected group were increased compared with those of control and MOLE-administered groups. Preadministration of MOLE to MTX-injected mice protected mice against altered MDA levels. The MTX-injected group showed significant decrease (*P* < 0.05) in total proteins, albumin, and globulin levels. Preadministration by MOLE protected significantly this decrease compared with MTX-injected mice (Figure 3).

### 3.4. Ameliorative Impacts of MOLE against MTX-Induced Changes on Levels of Inflammatory Cytokines (IL-1*β* and IL-6)

Injection of MTX significantly increased the serum levels of examined IL-1*β* and IL-6 (Table 5). MOLE administration alone decreased IL-1 and IL-6 levels. Preadministration of MOLE to the MTX-group maintained the levels of examined IL-1 and IL-6 within normal levels compared with the control group (Table 5).

### 3.5. Changes in the Expression of Antioxidants

**Figure 4:**
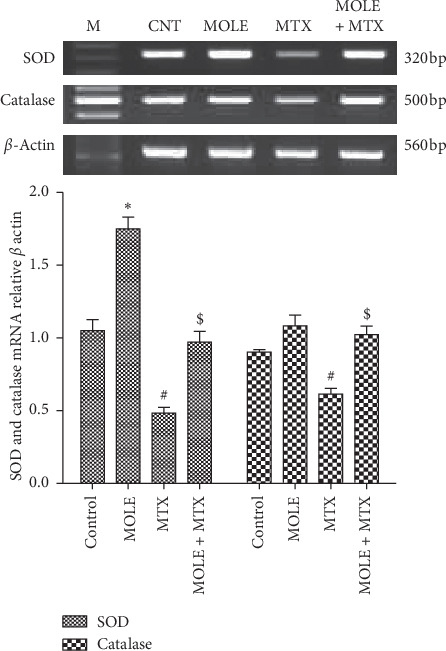
The ameliorative impact of MOLE on mRNA expression of SOD and catalase in MTX-treated mice. Graphic presentation of splenic mRNA levels by semiquantitative PCR analysis of SOD and catalase in different groups of mice after normalization with beta actin. ^*∗*^*P* < 0.05 versus control group; ^#^*P* < 0.05 versus control and MOLE groups; and ^$^*P* < 0.05 versus MTX-treated group.

The impact of MOLE on the SOD and catalase genes was examined. Mice were injected with MTX alone or after pretreatment with MOLE. SOD and catalase mRNA were downregulated in MTX-injected mice (Figure 4). Preadministration of MOLE recovered this downregulation compared with MTX-injected mice (Figure 4).

### 3.6. Changes in Cytokine Expression

**Figure 5:**
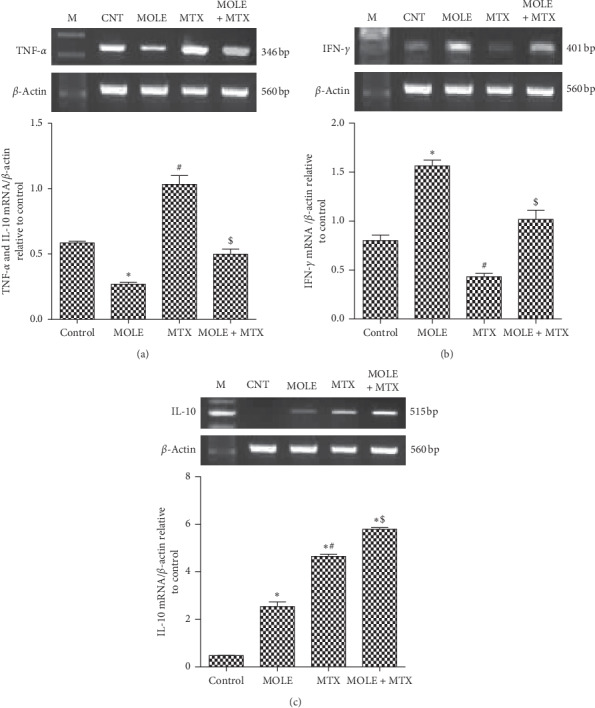
The ameliorative impact of MOLE on mRNA expression of TNF-*α*, IFN-*γ*, and IL-10 in MTX-treated mice. Graphic presentation of splenic mRNA levels by semiquantitative PCR analysis of TNF-*α*, IFN-*γ*, and IL-10 in different groups of mice after normalization with beta actin. ^*∗*^*P* < 0.05 versus control group; ^#^*P* < 0.05 versus control and MOLE groups; and ^$^*P* < 0.05 versus MTX-treated group.

Next, we examined the protective impact of MOLE administration on the expression of cytokines altered by MTX toxicity in the spleen.  Figure 5(a) shows that MTX upregulated the expression of TNF-*α*; in contrast, MOLE downregulated the TNF-*α* expression. Preadministration of MOLE to MTX-injected mice normalized the upregulation in TNF-*α* expression. Unlike TNF-*α* expression, the genetic expression of IFN-*γ* was upregulated by MOLE, while MTX downregulated it compared with that of control mice (Figure 5(b)). MOLE preadministration normalized and recovered IFN-*γ* to control levels. Of interest, the IL-10 expression was upregulated by MOLE and was more expressed in the MTX-injected group (Figure 5(c)). Surprisingly, preadministration of MOLE to the MTX group induced more additive expression for IL-10.

### 3.7. Changes in Apoptosis and Antiapoptosis Genes

**Figure 6:**
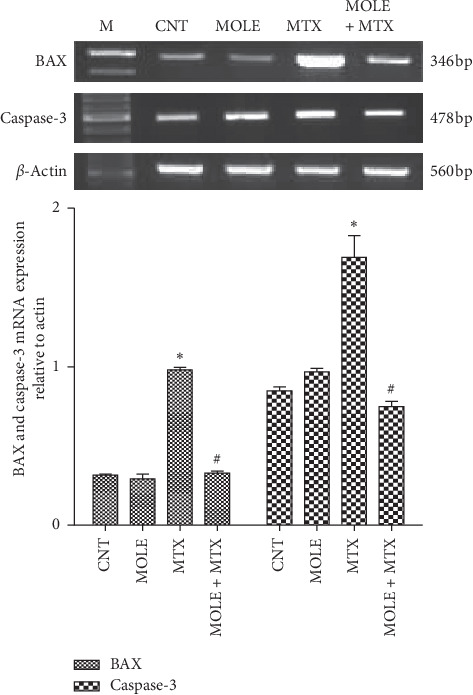
The ameliorative impact of MOLE on mRNA expression of BAX and caspases-3 in MTX-treated mice. Graphic presentation of splenic mRNA levels by semiquantitative PCR analysis of BAX and caspases-3 in different groups of mice after normalization with beta actin. ^*∗*^*P* < 0.05 versus control group; ^#^*P* < 0.05 versus control and MOLE groups; and ^$^*P* < 0.05 versus MTX-treated group.

**Figure 7:**
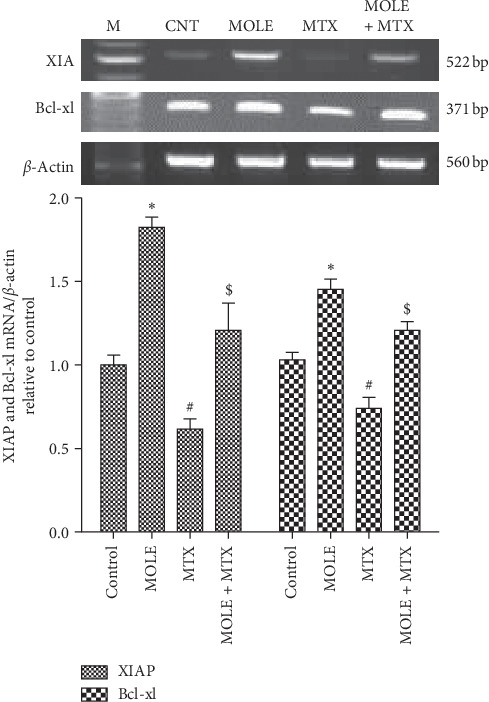
The ameliorative impact of MOLE on mRNA expression of XIAP and Bcl-xl in MTX-treated mice. Graphic presentation of splenic mRNA levels by semiquantitative PCR analysis of XIAP and Bcl-xl in different groups of mice after normalization with beta actin. ^*∗*^*P* < 0.05 versus control group; ^#^*P* < 0.05 versus control and MOLE groups; and ^$^*P* < 0.05 versus MTX-treated group.

Injection of MTX upregulated the genetic expression of BAX and caspase-3 (apoptosis associated genes) compared with that of control and MOLE administered mice (Figure 6). Preadministration of MOLE protected mice from reported changes in the BAX and caspase-3 expression altered by MTX injection. Of note, MOLE-administered mice upregulated the antiapoptotic genes, as MOLE upregulated the expression of XIAP and Bcl-xl (Figure 7). MTX-injected mice showed downregulation in the expression of XIAP and Bcl-xl compared with that of control and MOLE administered mice. Of interest, preadministration of MOLE to MTX-injected mice normalized the increase in apoptosis-associated genes and recovered the decrease in antiapoptosis genes reported in MTX-intoxicated mice (Figures 6 and 7).

### 3.8. Changes in NLRP3 and HO-1 Expression

**Figure 8:**
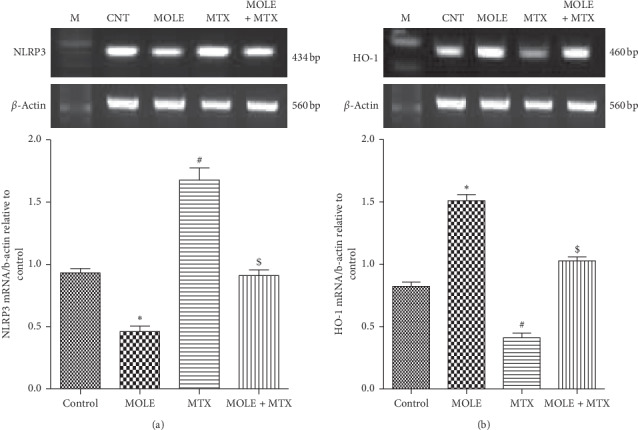
The ameliorative impact of MOLE on mRNA expression of NLRP3 and HO-1 in MTX-treated mice. Graphic presentation of splenic mRNA levels by semiquantitative PCR analysis of NLRP3 and HO-1 in different groups of mice after normalization with beta actin. ^*∗*^*P* < 0.05 versus control group; ^#^*P* < 0.05 versus control and MOLE groups; and ^$^*P* < 0.05 versus MTX-treated group.

Injection of MTX induced a general state of inflammation in spleen tissues as indicated by the upregulation in the expression of NLRP3 and downregulation in HO-1 compared with control and MOLE-administered groups (Figures 8(a) and 8(b)). The preadministration of MOLE for a week prior to MTX injection ameliorated the changes in NLRP3 and HO-1 expression compared with the MTX-injected group (Figure 8).

## 4. Discussion

The current study confirmed that dried leaves of MO are great source of polyphenol compounds, such as phenolic acids and flavonoids. Ingestion and intake of flavonoids protected patients against chronic diseases associated with oxidative stress [35]. MO leaves are good source of flavonoids. The flavonoids contained in MOLE are quercetin and kaempferol, in concentrations of 21.18 and 6.18, respectively, as reported here and by others [36]. As known, flavonoids such as quercetin are strong antioxidants, hypolipidemic, antidiabetic, and hypotensive and reduce oxidative stress and associated apoptosis [37]. Ferulic acid and resorcinol are the main phenolic compounds detected in our extracted MOL at a concentration of 61.09% and 7.46%, respectively. The phenolic compounds in MOL act as anti-inflammatory, antioxidant, and antiapoptotic factors [38].

Several reports concluded that MTX is widely used as an effective drug in chemotherapy and cancer treatment. But, it leads to some symptoms of direct toxicity [16, 39]. Moreover, it is the cause for second malignancy which is associated with several therapeutic treatments [40]. In this study, MTX-induced significant alteration in antioxidants and increased oxidative stress biomarkers were protected by preadministration of MOLE in the spleen. The toxic effects of MTX and other chemotherapy drugs are due to oxidative stress and the ROS formation [39].

As known, dietary antioxidants from herbal plants ameliorate the impacts of MTX chemotherapy by decreasing and/or preventing certain side effects related to MTX intoxication [11]. MTX increased the free radicals that are generated from both endogenous (cell activation and inflammation) and exogenous (environmental pollutants and chemotherapies) sources [41]. Literatures had implicated oxidative stress in MTX toxicity [41]. Oxidative stress mediated by MTX induced depletion in SOD, GSH, and CAT activities and induction of lipid peroxidation [42]. All these side effects were protected and maintained within normal ranges by preadministration of MOLE to the MTX-injected group (Tables 3 and 4 and Figures 3 and 4).

Previous works [42, 43] reported that MTX toxicity increased the inflammatory cytokine production and the ROS generation. Here, MTX induced proinflammatory responses, as it upregulated the expression of IL-1*β*, IL-6, TNF-*α*, and IL-10 and downregulated the IFN-*γ* expression. Administration of MOLE alone or preadministration to the MTX group protected and ameliorated the bad effects induced by MTX. MOLE improved the anti-inflammatory state by upregulation of different cytokine expressions. As known, MOLE is enriched with ferulic acid, quercetin, and kaempherol as polyphenolic compounds [44]. Ferulic acid, the main component of MOLE, is a phenolic compound with antioxidant, anti-inflammatory, antidiabetic, and organ-protective properties [45]. MOLE controlled the secretion of cytokines to achieve and control the balance between the pro- and counterinflammatory signaling pathways [46]. Therefore, the antioxidative and anti-inflammatory functions of MOLE reported hereby can be attributed to the phenolic compounds and flavonoid contained in MOLE.

In this study, the changes in expression of IFN-*γ*, TNF-*α*, and IL-10. IFN-*γ* were examined. These cytokines are secreted by lymphocytes and are involved in the expression of histocompatibility antigen and immune adjustment [47]. The interaction between IFN-*γ* and other proinflammatory cytokines can change the structure of cells and affects cells permeability [48]. In addition, MTX was reported to increase the secretion of proinflammatory cytokines (TNF-*α*, IL-1*β*, and IL-6) [48, 49]. These results reveal that the combined effects of MOLE as anti-inflammatory and antioxidant factor would be beneficial in the prevention of MTX-induced spleen toxicity. Therefore, MOLE induced potentiation in IL-10 expression to control and regulate the expression of TNF-*α*, IFN-*γ*, and IL-10 [1, 47, 50].

Bax and Bcl-2 genes are members of Bcl-2 family that control cell susceptibility against destruction, degradation, and apoptosis [51]. Bax is known as proapoptotic protein activated by oxidative stress and inflammatory cytokines [52]. Unlike Bax, Bcl-2 blocks and antagonizes apoptosis through caspase activation [53]. Intoxicated splenic cells by methotrexate upregulated Bax and caspase-3 and downregulated the XIAP and Bcl-xl expression. All were ameliorated when MOLE were preadministered to MTX-injected mice. Preadministration of MOLE prevented MTX-induced cell death via its antioxidant and anti-inflammatory effects. MOLE downregulated Bax and caspase-3 and upregulated Bcl-xl in the spleen of MTX-intoxicated mice, confirming the potential antiapoptotic impact of MOLE. In parallel, XIAP (inhibitor of apoptosis) stops apoptotic cell death. The current finding confirmed the potentiation effect of MOLE as an antiapoptotic factor through its effect on Bcl-xl and XIAP expression as antiapoptotic genes.

**Figure 9:**
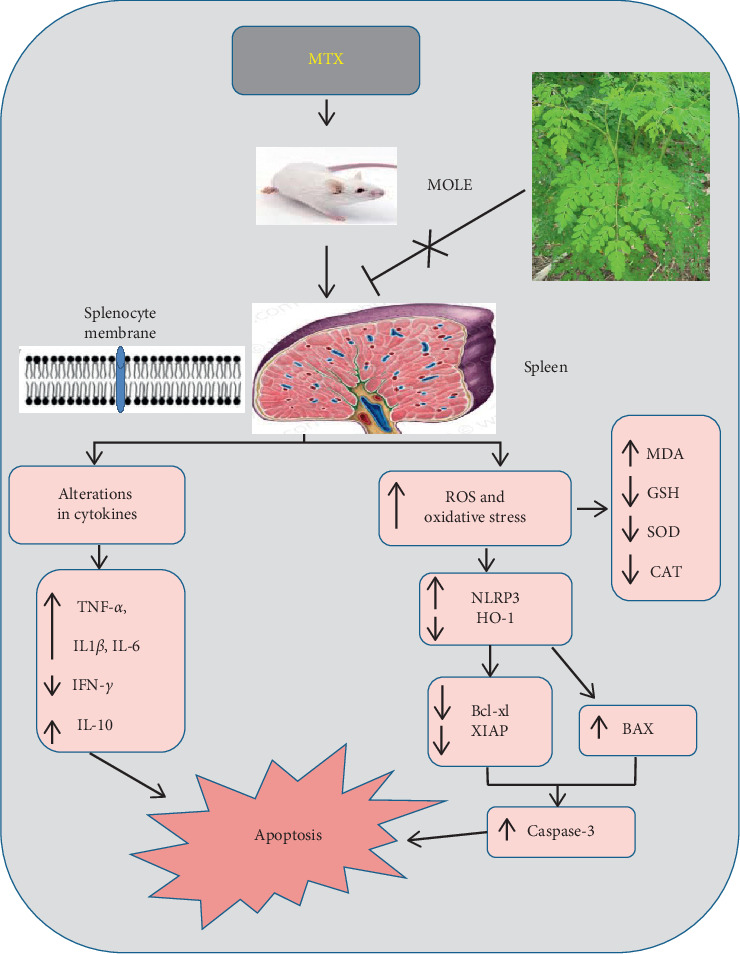
Graphical abstract representing the spleen-protective effects of MOLE on MTX-induced splenic intoxication.

To explore the mechanisms by which the effect of MOLE on MTX induced spleen toxicity was achieved, we examined the expression pattern of NLRP3 and HO-1. The fact that ROS elicits NLRP3 activation might provide an explanation of the anti-inflammatory potential of HO-1. HO-1 is a protective gene, incorporated in the regulation and production of anti-inflammatory, antioxidants, and antiapoptotic metabolites [54]. HO-1 prevents inflammation. ROS contributed and induced inflammasome activation. Inflammasome especially NLRP3 regulates inflammation in different tissues by activating some cytokines such as IL-1*β* and IL-18 [55]. HO-1 regulates the inflammasome (NLRP3) expression as reported here and by others [56]. We reported that MTX downregulated HO-1 and upregulated NLRP3 expressions, though MOLE preadministration markedly enhanced the HO-1 expression and lowered the NLRP3 expression. Here, MOLE upregulated HO-1 to restore the increase in the NLRP3 expression and its associated inflammatory cascade [57]. The collective protective impacts of MOLE against MTX-induced spleen toxicity are summarized in a graphical figure (Figure 9).

In conclusion, current findings demonstrate that MOLE exerts protective impacts against MTX-induced spleen toxicity and oxidative stress. The protective impacts of MOLE is mediated through the regulation of anti-inflammatory, antioxidant, and antiapoptotic signaling pathways. Therefore, MOLE is effective as a therapeutic agent for splenic oxidative stress relief and treatment.

## Figures and Tables

**Table 1:** PCR conditions of examined genes in mice.

Gene	Product size (bp)	Annealing temperature	Primer sequence
BAX	483	53°C, 1 min	Forward 5′-acacctgagctgaccttggagca-3′ Reverse 5′-agacacagtccaaggcagtggga-3′

Caspase-3	478	53°C, 1 min	Forward 5′-acgcagccaacctcagagagaca-3′ Reverse 5′-ttgtgcgcgtacagcttcagcat-3′

XIAP	522	59°C, 1 min	Forward 5′-gggtgggtcttagaggggcttat-3′ Reverse 5′-acaagccatatacaccaggcgtc-3′

Bcl-XL	371	54°C, 1 min	Forward 5′-ccactggccacagcagcagttt-3′ Reverse 5′-aaaagtgtcccagccgccgtt-3′

SOD	320	55°C, 1 min	Forward 5′-gtcctttcctgcggcgcctt-3′ Reverse 5′-cgccgggccaccatgtttct-3′

Catalase	500	58°C, 1 min	Forward 5′-ggcccctcctcgttcaggatgt-3′ Reverse 5′-gccattcatgtgccggtgacca-3′

HO-1	460	55°C, 1 min	Forward 5′-tcaggtgtccagagaaggcttta-3′ Reverse 5′-aggtgtcatctccagagtgttca-3′

NLRP3	434	56°C, 1 min	Forward 5′-agctgctggcctgacccaaa-3′ Reverse 5′-agccccgtgcacacaatcc-3′

TNF-*α*	346	56°C, 1 min	Forward 5′-aactagtggtgccagccgat-3′ Reverse 5′-cttcacagagcaatgactcc-3′

IL-1-*β*	409	58.5°C, 1 min	Forward 5′-ccgtggaccttccaggatga-3′ Reverse 5′-gatccacactctccagctgc-3′

IL-10	515	59°C, 1 min	Forward 5′-agagcaaggcagtggagcaggt-3′ Reverse 5′-aagggccctgcagctctcaagt-3′

INF-*γ*	401	58.5°C, 1 min	Forward 5′-cccacaggtccagcgccaag-3′ Reverse 5′-gctgtcccccacccccagat-3′

*β*-actin	560	56°C, 1 min	Forward 5′-agatccacaacggatacatt-3′ Reverse 5′-tccctcaagattgtcagcaa-3′

PCR cycle of respective genes are shown, while temperature and time of denaturation and elongation steps of each PCR cycle are 94°C, 30 s and 72°C, 60 s, respectively; annealing temperature was indicated for each gene in the table.

**Table 2:** Phenolic compounds in *Moringa oleifera* leaf extract (MOLE).

Integration results							
No.	Peak name	Retention time (min)	Area (mAU *∗* min)	Height (mAU)	Relative area (%)	Relative height (%)	Amount (ppm)
n.a.	Coumarin	n.a.	n.a.	n.a.	n.a.	n.a.	n.a.
1	Ferulic acid	2.393	5.213	107.679	31.78	61.09	78.6167
2	Resorcinol	2.443	0.278	13.154	1.70	7.46	0.2834
3	Quercetin	3.213	7.424	37.330	45.27	21.18	94.1503
4	Kaempferol	3.920	2.851	10.892	17.38	6.18	35.4734

n.a.: not detectable.

**Table 3:** Ameliorative effects of MOLE against MTX-induced oxidative stress in splenic mice homogenate.

	Control	MOLE	MTX	MOLE + MTX
MDA (nmol/g tissue)	14.78 ± 1.2	13.7 ± 3.3	46.3 ± 2.5^#^	19.3 ± 1.87^$^
SOD (U/g tissue)	9.5 ± 2.2	14 ± 2.8^*∗*^	6 ± 0.3^#^	10.1 ± 1.1^$^
GSH (nmol/100 mg)	65 ± 5.4	75 ± 6.9^*∗*^	22.4 ± 4.8^#^	44.1 ± 5.7^$^

Values are means ± SEM for 7 different mice per each experiment. Values are statistically significant at ^*∗*^*P* < 0.05 Vs. control; ^#^*P* < 0.05 Vs control and moringa groups and ^$^*P* < 0.05 Vs. MTX group. SOD: superoxide dismutase; MDA: malondialdehyde; MOLE: *Moringa oleifera* leaf extract; MTX: methotrexate.

**Table 4:** Ameliorative effects of MOLE on serum antioxidants levels in MTX-injected mice.

	Control	MOLE	MTX	MOLE + MTX
IL-1*β* (pg/ml)	162 ± 6.8	132.3 ± 2.3^*∗*^	260 ± 28.2^#^	163.3 ± 14.3^$^
IL-6 (pg/ml)	66.3 ± 8.8	45.3 ± 1.2^*∗*^	208 ± 31.3^#^	92 ± 3.08^$^

Values are mean ± standard error (SEM) for 7 different mice per each treatment. Values are statistically significant at ^*∗*^*P* < 0.05 vs control; ^#^*P* < 0.05 vs control and MOLE groups and ^$^*P* < 0.05 vs MTX group. SOD: superoxide dismutase; MDA: malondialdehyde; GSH: reduced glutathione; MOLE: *Moringa oleifera* leaf extract; MTX: methotrexate.

**Table 5:** Ameliorative effects of MOLE against MTX-induced changes on serum levels of IL-1*β* and IL-6.

	SOD (U/ml)	GSH (nmol/l)	Catalase (U/l)	MDA (nmol/ml)
Control	3.4 ± 0.15	3.5 ± 0.3	179 ± 8.8	14.01 ± 1.5
MOLE	3.9 ± .08	4.6 ± 0.2	208.7 ± 5.1^*∗*^	12.7 ± 1.5
MTX	1.8 ± 0.3^#^	2.4 ± 0.08^#^	138.2 ± 2.9^#^	38.3 ± 1.8^#^
MOLE + MTX	2.9 ± 0.2^$^	3.5 ± 0.1^$^	170.7 ± 5.1^$^	20.4 ± 1.2^$^

Values are mean ± SEM for 7 different mice per each experiment. Values are statistically significant at ^*∗*^*P* < 0.05 Vs. control; ^#^*P* < 0.05 Vs control and MOLE groups and ^$^*P* < 0.05 Vs. MTX group.

## Data Availability

The data used to support the findings of this study are available from the corresponding author on reasonable request
